# Fabrication of Hexagonal Microlens Arrays on Single-Crystal Silicon Using the Tool-Servo Driven Segment Turning Method

**DOI:** 10.3390/mi8110323

**Published:** 2017-10-30

**Authors:** Mao Mukaida, Jiwang Yan

**Affiliations:** Department of Mechanical Engineering, Faculty of Science and Technology, Keio University, 3-14-1 Hiyoshi, Kohoku-ku, Yokohama 223-8522, Japan; mukaida@keio.jp

**Keywords:** single-crystal silicon, ultraprecision cutting, slow tool servo, microlens array, Infrared optics, ductile machining

## Abstract

Single-crystal silicon microlens arrays are increasingly required in advanced infrared optics. In this study, the authors attempted to fabricate hexagonal microlens arrays, which offer high optical efficiency, on a single-crystal silicon wafer using diamond turning. A tool-servo driven segment turning method was proposed to reduce the dynamic error of the machine tool induced by lenslet edges during lens array cutting. From the results of both cutting experiments and theoretical analysis of the machine tool dynamic error, it was demonstrated that the segment turning method reduced significantly the dynamic errors and led to high form accuracy. As a result, sharp edges among the lenslets were generated precisely and microlens arrays with a form error of ~300 nm peak-to-valley and surface roughness of ~5 nmSa, which meets the requirements of infrared optical systems, were successfully fabricated. The subsurface damage, such as the amorphization of silicon, caused by machining was also reduced.

## 1. Introduction

In recent years, infrared (IR) optical systems are increasingly demanded in many fields such as security and remote sensing. Single-crystal silicon is a typical substrate material for IR optics because of its high transmittance in the IR region. Although spherical and aspherical lenses of single-crystal silicon have been used in IR optical systems so far [1,2,3], the silicon lens with its more complex shapes is in demand for future IR optical systems. For example, a microlens array can focus light on photodiodes in image sensors and homogenizers [4,5,6]. Microlens arrays have been widely used in optical systems but currently most of them are made of glass and plastics, which are used only for visible or near-infrared lights. To date, there is very little literature on the fabrication of microlens arrays for IR systems.

In addition, silicon microlens arrays are also useable as micro mirrors for laser beam shaping [4]. Compared with other mirror materials, such as metal-coated glass substrates, single-crystal silicon is more suitable because of its high thermal conductivity. Another possible use of silicon microlens arrays is as the molds for press molding of glass micro optics [7].

A few methods have been attempted for fabricating microlens arrays on silicon. Most of those methods are based on lithographic processes such as chemical etching [8,9,10] and laser assisted etching [4,5]. However, the available lens geometries are very limited and it is difficult to ensure the form accuracy of the lens arrays.

On the other hand, mechanical machining technologies, such as ultraprecision cutting, have the advantages of high freedom of lens geometry, high form accuracy and low surface roughness [11]. In recent years, diamond turning using tool servos has enabled machining freeform surfaces by synchronizing the tool movement with the spindle rotation [11,12,13,14,15], which reduces machining time significantly compared to other cutting methods such as micro milling. There are two kinds of tool servos: a slow tool servo (STS) driven by a table of the machine tool and a fast tool servo (FTS) driven by a piezoelectric actuator in addition to the machine tool itself. Normally, an FTS is suitable for generating small-amplitude microstructures on flat or axially symmetrical surfaces; while an STS enables machining large-amplitude surface structures and high aspect ratio freeform surfaces. Also, STS systems are preferable for economic reasons because no extra machine add-ons are necessary for tool drive apart from the machine tool, unlike the FTS.

In a previous paper of the present authors, we succeeded in machining circular dimples on single-crystal silicon by STS diamond turning [16]. Independent circular lens dimples with high form accuracy were generated on silicon by ductile machining. To further increase the optical function, however, closely connected gapless microlens arrays are required. In this study, fabrication of hexagonal microlens arrays on single-crystal silicon was attempted. Hexagonal microlens arrays offer a fill factor of 100% and achieve higher light efficiency than circular lens arrays [17].

However, hexagonal microlens arrays have many sharp edges at the boundaries among the lenslets, which makes the STS diamond turning very difficult because there is a significant dynamic error caused by the excessive acceleration of machine tables [18]. For this reason, up to date, STS diamond turning has only been used for machining continuous surfaces without edges [12,19,20,21]. A few researchers reported machining of microlens arrays using an STS [22,23], but the achieved form accuracy was low and no consideration was given to the edge-induced form error.

In this paper, a new STS diamond turning method, namely, segment turning method, for machining gapless hexagonal microlens arrays is proposed to improve lens form accuracy and edge generation. First, the effectiveness of the segment turning method will be experimentally evaluated by comparing with the conventional STS turning method in cutting hexagonal microlens arrays on an aluminum alloy. Ductile machining of hexagonal microlens arrays will then be attempted on single-crystal silicon by the segment turning method. The objective is to realize ductile machining of hexagonal microlens arrays on single-crystal silicon with low subsurface damage.

## 2. Mechanism of Segment Turning

**Figure 1:**
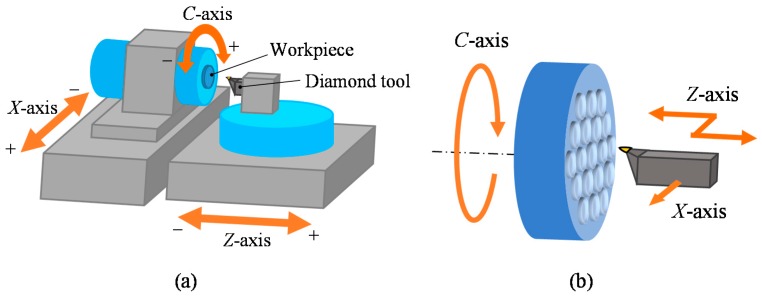
Schematic of slow tool servo (STS) diamond turning: (**a**) lathe configuration; and (**b**) freeform machining.

**Figure 2:**
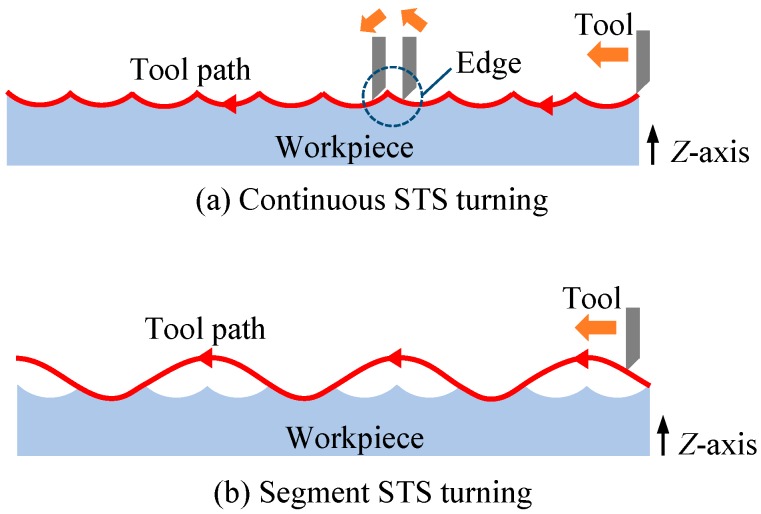
Schematics of tool paths for two STS turning methods to generate a microlens array.

Figure 1a shows a schematic diagram of an ultraprecision diamond turning lathe having an STS system and Figure 1b shows freeform surface machining by using the STS system. In STS turning, the *Z*-axis position of the tool is synchronized with the *C*-axis rotation of the workpiece, which means *Z* coordinate is a function of *X* and *C*. Conventionally, when machining concave microlens arrays, all lenslets are continuously machined in a single turning cycle, as shown in Figure 2a. Continuous STS turning leads to excessive *Z*-axis acceleration at the sharp-edge boundaries among the lenslets and this causes dynamic errors of the machine tool. In this study, segment STS turning is proposed, as shown in Figure 2b. In this method, the lenslets are divided into multiple groups where the lenslets in each group are separated from each other and each group of lenslets are machined in a single turning cycle. In this way, the sudden change in direction of the tool path is avoided, which reduces the *Z*-axis acceleration. It is expected that this method improves the motion accuracy of the machine tool and in turn, the resulting lens accuracy.

## 3. Effectiveness Verification of Segment turning

### 3.1. Lens Design and Tool Path Generation

**Figure 3:**
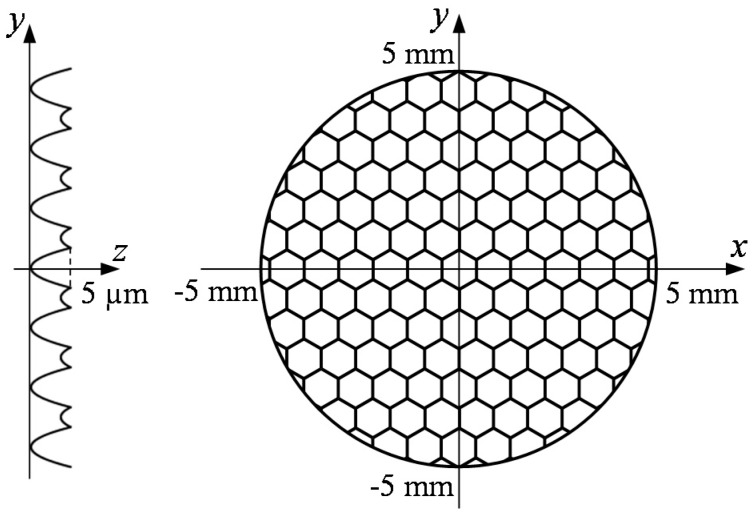
Lens array design for cutting experiments of comparison between segment turning and continuous turning.

To investigate the effectiveness of segment turning method, hexagonal microlens arrays, which consist of sharp edges among lenslets, were machined by continuous STS turning and segment STS turning, respectively and the results were compared. Figure 3 shows the designed shape of a microlens array. The workpiece was 10 mm in diameter and each lenslet had a concave spherical surface with a radius of 25 mm and a hexagonal shape with a side length of 500 μm. The lenslet sag was 5 μm. The Cartesian coordinates (*x*, *y*, *z*) were set to the surface of the workpiece, while the cylindrical coordinates (*X*, *Z*, *C*) were set to the machine tool. Then, these coordinates have the following relationships:

(1) x=XcosC

(2) y=XsinC

(3) z=Z

**Figure 4:**
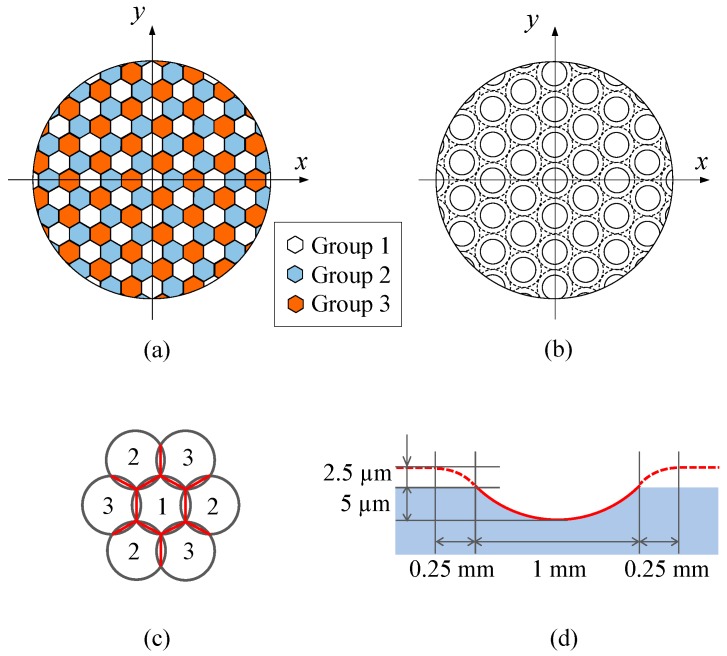
Tool path generation for segment turning: (**a**) schematic of segments; (**b**) dimples generated in a single cycle; (**c**) generation of hexagonal shape; and (**d**) tool path for each lenslet.

Tool path was then generated using the optics machining software DIFFSYS (AMETEK Precitech Inc., Keene, NH, USA). The software enables tool radius compensation when generating tool paths. Figure 4a shows the distribution of divided lenslet groups in segment turning. In the present test cut, the whole lens array was divided into three groups which were cut sequentially. In a single turning cycle, circular lenslets were cut as shown in Figure 4b and then hexagonal shape was formed by overlapping the circular lenslets as shown in Figure 4c. In addition, the outer region of each lenslet had an approach zone for the tool as described by the dashed circle in Figure 4b and the dashed line in Figure 4d. The approach zone was designed to make the tool path smooth without sharp turning points. In both continuous turning and segment turning, control points (a control point is a member of a set of points used to determine the shape of a spline curve) were calculated at a constant angular step (2°) on the spiral tool path trajectory around the spindle axis. Linear interpolation was adopted among adjacent control points.

### 3.2. Cutting Experiments

An ultraprecision diamond turning lathe Precitech Nanoform X (AMETEK Precitech Inc.) having an STS system was used in the experiments. A single-crystal diamond tool with a nose radius of 0.1 mm, a rake angle of 0° and a relief angle of 6° was used. Cutting experiments were conducted on an aluminum alloy A5056, on which tool movement can be well transferred. Oil mist was used for lubrication and cooling during cutting. The cutting parameters are summarized in Table 1. In order to compare the two methods in terms of cutting speed and machining time, the spindle rotation rate (*N*) of continuous turning was set to two levels: one is the same as that of segment turning (45 rpm) and the other is one-third of that of segment turning (15 rpm), respectively.

### 3.3. Lens Topographical Error

**Figure 5:**
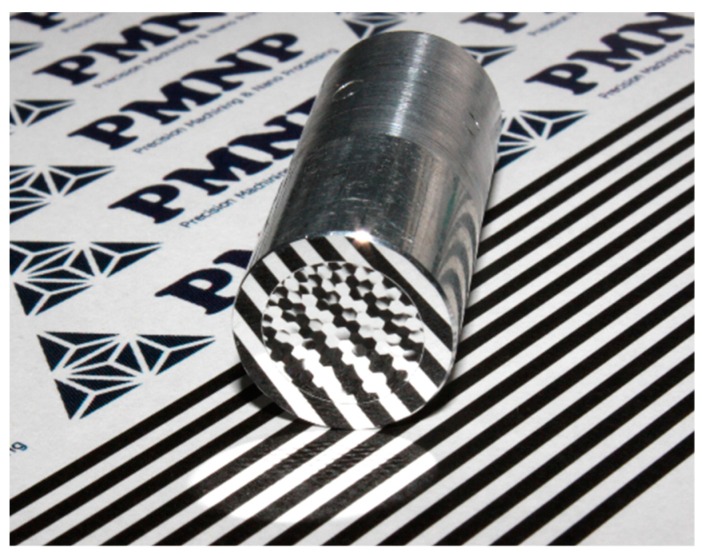
Photograph of a hexagonal microlens array on aluminum alloy machined by segment STS turning at *N* = 45 rpm.

**Figure 6:**
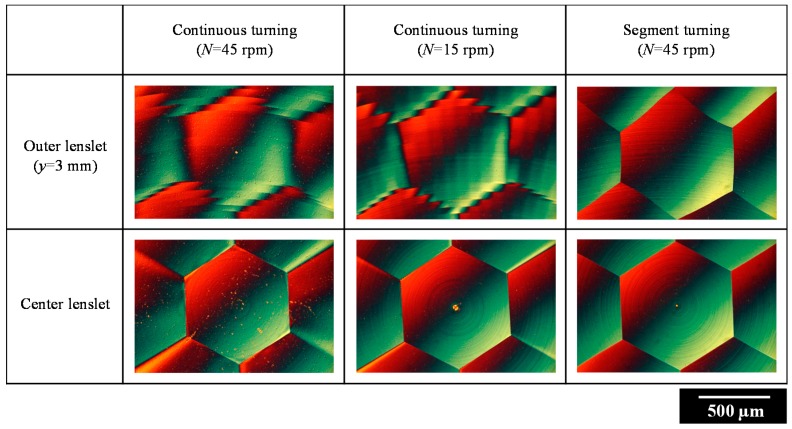
Differential interference contrast microscope images of lenslets machined by continuous turning at different spindle rotation rates, as well as by segment turning.

Figure 5 shows a photograph of a microlens array sample machined by segment STS turning at *N* = 45 rpm, which has a mirror finish. The sample surface was then observed using a differential interference contrast microscope. Figure 6 shows microscope images of lenslets machined by continuous turning at different spindle rotation rates, as well as by segment turning. Two groups of images are shown: the lenslets around the workpiece center and those located at an outer region around *x* = 0 mm and *y* = 3.0 mm. From these images, it is clear that the lens edges got blunt and disordered in continuous turning as the distance from the workpiece center increased. In contrast, very sharp edges were generated across the workpiece surface machined by segment turning.

**Figure 7:**
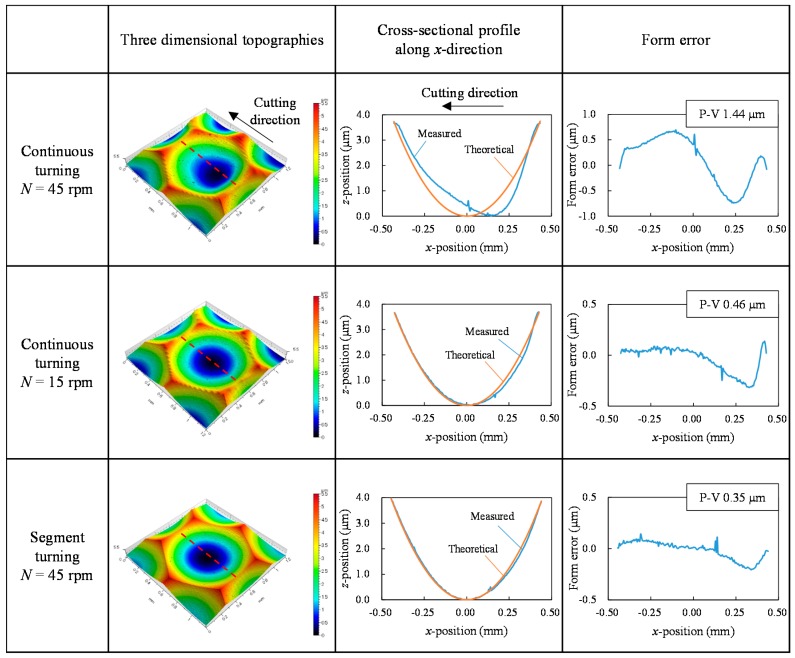
Three-dimensional topographies and cross-sectional profiles of lenslets the centers of which are located at *x* = 0 mm, *y* = 1.5 mm.

Next, the surfaces of the machined lens dimples were measured in detail using a laser-probe profilometer NH-3SP (Mitaka Kouki Co., Ltd., Tokyo, Japan). Figure 7 shows three-dimensional topographies of lenslets the center of which are located at *x* = 0 mm, *y* = 1.5 mm in Figure 3 and cross-sectional profiles of lenslets measured along *x*-axis through the lenslet centers as indicated by the red dashed lines in the three-dimensional topographies. By comparing the theoretical profiles and the measured profiles, form error distributions were obtained and plotted in Figure 7. The results show that the peak-to-valley (P-V) value of the cross-sectional form error in segment turning was 0.35 μm, 24% of that in continuous turning (1.44 μm) at the same spindle rotation rate (*N* = 45 rpm) and 76% of that in continuous turning (0.46 μm) for the same machining time but at a lower spindle rotation rate (*N* = 15 rpm). This means that segment turning offers higher productivity than continuous turning. In addition, a large form error was found in the right side of the plots. Since the cutting direction was in the *x*-axis negative direction, the form error increased just after passing lenslet edges. Thus, it can be said that lenslet edges induced significant form errors and these form errors can be effectively reduced in segment turning.

### 3.4. Measurement of Machine Tool Dynamic Error

**Figure 8:**
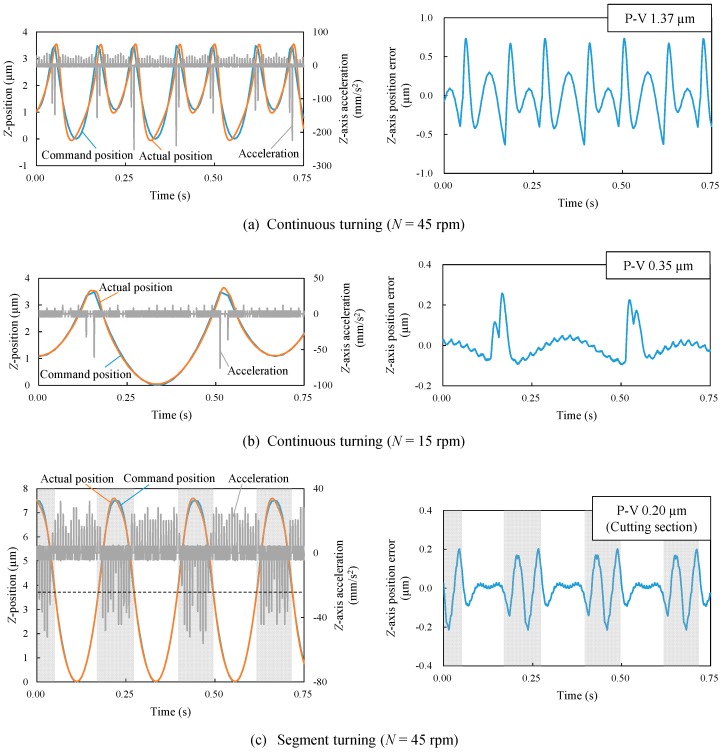
Plots of command positions, actual positions and accelerations (left-side graphs), as well as position errors (right-side graphs) for continuous turning and segment turning. (**a**) Continuous turning (*N* = 45 rpm); (**b**) continuous turning (*N* = 15 rpm); (**c**) segment turning (*N* = 45 rpm).

To investigate the dynamic errors of the machine tool, the *Z*-axis position was measured by using the real-time process monitoring system equipped in the machine tool itself and compared with command position. Then, the *Z*-axis position error was calculated from the difference between the command position and the actual position and the *Z*-axis acceleration was calculated from the change of the actual position, respectively. Figure 8 shows the plots of command positions, actual positions and accelerations in the left-side graphs and the plots of position errors in the right-side graphs. In the figure, the measurements were performed during a period of 0.75 s after the tool passes the point *X* = 1.5 mm in order to compare with the profile error shown in Figure 7.

In continuous turning at *N* = 45 rpm, the absolute value of acceleration at the lenslet edges was so large that it led to a significant delay in the *Z*-axis motion of the machine table (1.37 μm P-V). When the spindle rotation rate is decreased to *N* = 15 rpm, the acceleration decreased too, causing a decrease in the *Z*-axis position error to 0.35 μm P-V. However, in segment turning at *N* = 45 rpm, the acceleration was reduced by a factor of five compared to that in continuous turning at the same spindle rotation rate, which accordingly reduced position error of cutting section (*Z* ≤ 3.75 μm) to 0.20 μm P-V. The trend of *Z*-axis position error was almost the same as that of the lens form error shown in Figure 7, which means the lens form error was mainly caused by the dynamic error of the machine tool. Figure 8 demonstrates strongly that even if the *Z*-axis acceleration can be reduced by decreasing the spindle rotation rate in continuous turning, it is still significantly higher than that in segment turning.

**Figure 9:**
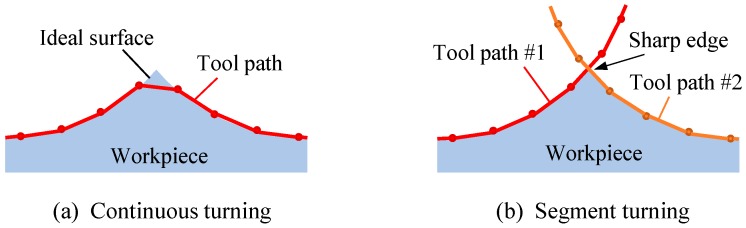
Effect of linear interpolation on lens edge formation for (**a**) continuous turning and (**b**) segment turning. (**a**) Continuous turning; (**b**) segment turning.

**Figure 10:**
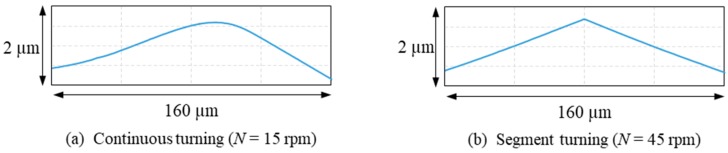
Magnified cross-sectional profiles of lens edges measured using a white light interferometer, showing a blunt edge for (**a**) continuous turning and a sharp edge for (**b**) segment turning. (**a**) Continuous turning (*N* = 45 rpm); (**b**) segment turning (*N* = 45 rpm).

An important factor affecting lens edge formation is interpolation. In STS turning, a freeform surface is generated by calculating coordinates of a finite number of control points on the objective surface and interpolating between these points. Linear interpolation and spline interpolation are two major methods for interpolation. Spline interpolation is suitable for making a smooth tool path, but cannot be used to generate sharp edges. To generate a sharp edge by continuous STS turning, linear interpolation is necessary. However, as shown in Figure 9a, a sharp edge cannot be formed in continuous turning when the number of control points is not adequate to envelope the edge through linear interpolation. In segment turning, however, the tool path is not affected by interpolation method, as shown in Figure 9b. This greatly improves the edge accuracy. The differences in tool paths at lenslet edges between continuous turning and segment turning can be confirmed in the left-side graphs of Figure 8a–c. In addition, Figure 10 shows magnified cross-sectional profiles of lens edges at *y* = 0.75 mm measured using a white light interferometer Talysurf CCI1000 (AMETEK Taylor Hobson Ltd., Leicester, UK). In continuous turning (Figure 10a), the edge is very dull, with a radius of several hundred microns. In segment turning (Figure 10b), however, the edge becomes so sharp that the radius of which is hard to identify even at the available magnification of the white light interferometer used in this study.

### 3.5. Modelling of Machine Tool Dynamic Error

**Figure 11:**
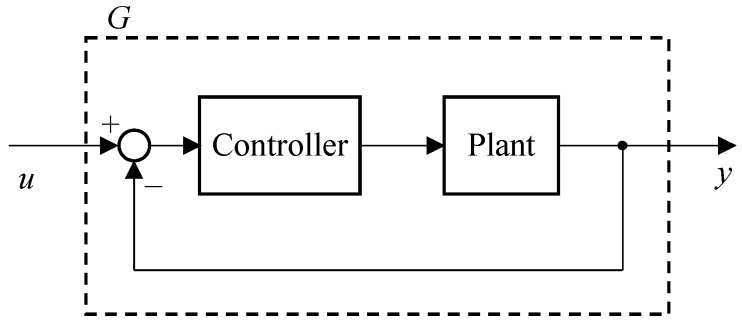
Block diagram of the feedback control system model for an STS system.

In order to predict the dynamic errors caused by an STS system in freeform surface generation, it is important to establish a theoretical model for the control system. The actual control system of the STS machine tool might be very complex with a complicated transfer function [24]. In this study, for simplicity, the STS system was modeled as an open-loop system described by transfer function *G* according to the direct approach [25], as shown in Figure 11.

**Figure 12:**
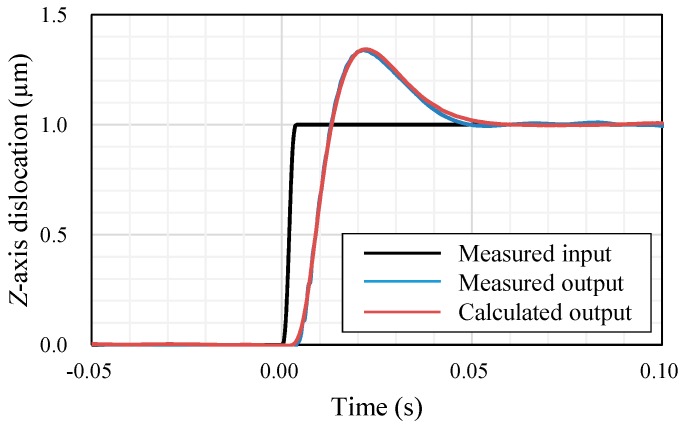
Measured and calculated step responses of the STS system.

To identify the transfer function *G*, the actual step response of the STS system was experimentally measured. A *Z*-axis displacement of 1 μm at a velocity of 1 mm/s was input to the machine tool as a step input and the output was measured by the real-time process monitoring system of the machine. The results are shown in Figure 12. Then, the transfer function *G* is assumed to be an ARX model as follows [25]:

(4) $$y\left(k\right)+a_{1}y\left(k-1\right)+\cdots +a_{n_{a}}y\left(k-n_{a}\right)=b_{1}u\left(k-1\right)+\cdots +b_{n_{b}}y\left(k-n_{b}\right)+w\left(k\right)$$

(5) $$A\left(q\right)=1+a_{1}q^{-1}+\cdots +a_{n_{a}}q^{-n_{a}}$$

(6) $$B\left(q\right)=b_{1}q^{-1}+\cdots +b_{n_{b}}q^{-n_{b}}$$

(7) $$y\left(k\right)=\frac{q^{-d}B\left(q\right)}{A\left(q\right)}u\left(k\right)+e\left(k\right)=G\left(q\right)u\left(k\right)+e\left(k\right)$$

where *u*(*k*) is input and *y*(*k*) is output at time *k*, *q* is shift operator and *e*(*k*) is disturbance at time *k*. Thus, the transfer function *G* can be determined by *n_a_*, *n_b_*, *d* and *a*_1_ … *a_na_*, *b*_1_ … *b_nb_*. These parameters were identified by least squares method fitting using the MATLAB System Identification Toolbox as step response of the identified transfer function meets the step response experimentally measured. Identified parameters are summarized in Table 2. The calculated step response with these parameters is also shown in Figure 12 and it is confirmed that the calculated result matches well with the experiments.

**Figure 13:**
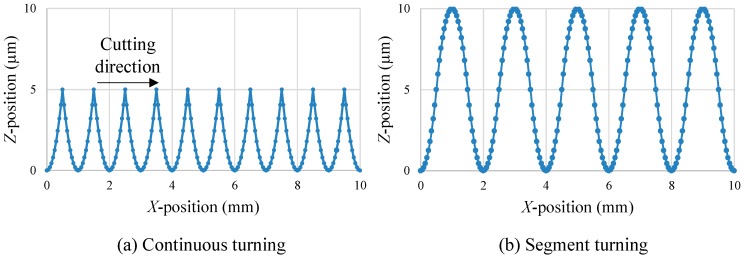
Input tool paths for calculating machine tool position errors when cutting concave spherical lenslets. (**a**) Continuous turning; (**b**) segment turning.

Then, the model was used to calculate dynamic errors of various tool paths for microlens array machining. Two typical input tool paths as shown in Figure 13 were used to generate lenslets having concave spherical surfaces with a depth of 5 μm and a pitch of 1 mm. Control points were set at an interval of 50 μm along *X*-direction and linear interpolation was used. The tool path of segment turning included non-cutting sections between lenslets, the shape of which was the reversed lenslet shape. Cutting speed was set to 7 mm/s and 2.3 mm/s on the assumption of turning at spindle rotation rates *N* = 45 rpm and *N* = 15 rpm by a distance of 1.5 mm from the spindle rotation center. These conditions correspond to those of position error measurement shown in Figure 8.

**Figure 14:**
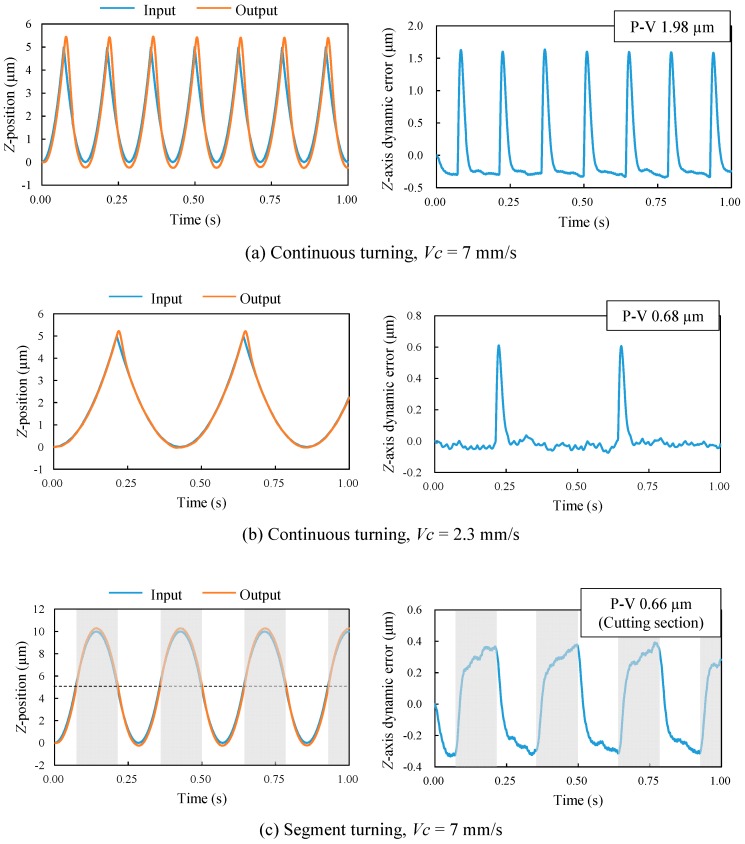
Calculated *Z*-axis positions (left-side graphs) and position errors (right-side graphs) for continuous turning and segment turning using an STS system. (**a**) Continuous turning, *Vc* = 7 mm/s; (**b**) continuous turning, *Vc* = 2.3 mm/s; (**c**) segment turning, *Vc* = 7 mm/s.

Figure 14 shows results of calculated *Z*-axis positions and position errors. In continuous turning, very large position errors occur at sharp edges even if the cutting speed is low. In segment turning, however, the P-V value of the position error is smaller than that in continuous turning; 33% of that in continuous turning at the same cutting speed (*Vc* = 7 mm/s) and 97% of that at the one-third cutting speed (*Vc* = 2.3 mm/s). Under the same conditions, the measured P-V value of position error of segment turning was 15% and 57%, respectively, of that in continuous turning. Larger dynamic errors in the calculated models might be due to the control system simplification. In addition, the ultraprecision machine tool we used in this study had an STS control system involving adaptive control, where the control system was continuously optimized during machining and thus the dynamic errors were suppressed effectively.

The distribution of the calculated position error had similar tendency as that of the measured results. From these facts, it might be said that the calculated results are comparable to the measured results and machine tool position errors can be approximately estimated by the prediction model used in this study. This model can help to generate tool path and decide machining parameters for non-rotationally symmetric surfaces.

The findings from the present study demonstrated that by using the segment turning method, an ultraprecision machine tool equipped with an STS system can be directly used for fabricating high-precision freeform and structured surfaces having sharp edges, without need for introducing machine add-ons such as FTS and so on. This will greatly extend the application fields of the STS-driven ultraprecision machine tools in advanced manufacturing industries for optics, optoelectronics and micromechanical elements.

## 4. Fabrication of Hexagonal Microlens Array on Single-Crystal Silicon

### 4.1. Experimental Procedure

**Figure 15:**
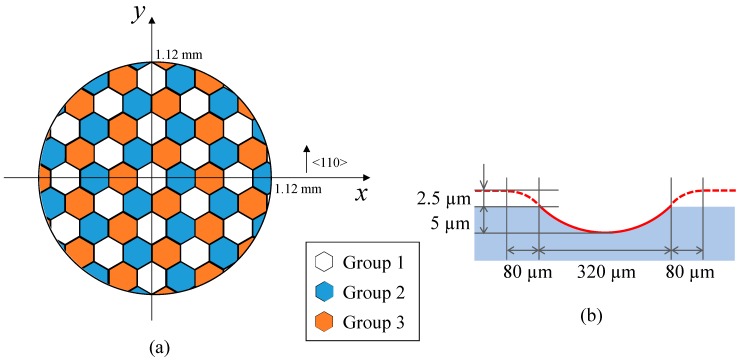
Designed shape of silicon microlens array and tool path for its fabrication: (**a**) schematic of lenslets segment; (**b**) tool path for each lenslet.

Hexagonal microlens arrays were machined on a single-crystal silicon (001) wafer using the segment STS turning method. The designed shape of the microlens array is shown in Figure 15, where the crystal orientation of silicon wafer is also indicated. Each lenslet has a concave spherical surface with a curvature radius of 2.563 mm and a hexagonal shape with a side length of 160 μm. The lenslet sag is 5 μm. The whole lens array was divided into three groups of lenslets, as described in Figure 15a. The outer region of the lenslets had an approach zone as described by the dashed line circle in Figure 15b.

The ultraprecision lathe Nanoform X (AMETEK Precitech Inc., Keene, NH, USA) having an STS system was used for the silicon cutting experiments as well. A single-crystal diamond tool with a nose radius of 1 mm, a rake angle of −30° and a flank angle of 36° was used. Experimental conditions are summarized in Table 3. Feed rate *f* was determined based on the results of dimple cutting experiments as reported in Ref. [16]. In this experiment, control points were calculated at a constant angular step of 1° on the spiral tool path trajectory around the spindle axis and spline interpolation was adopted among adjacent control points to describe the ideal lenslet surface more precisely.

### 4.2. Lens form Accuracy

**Figure 16:**
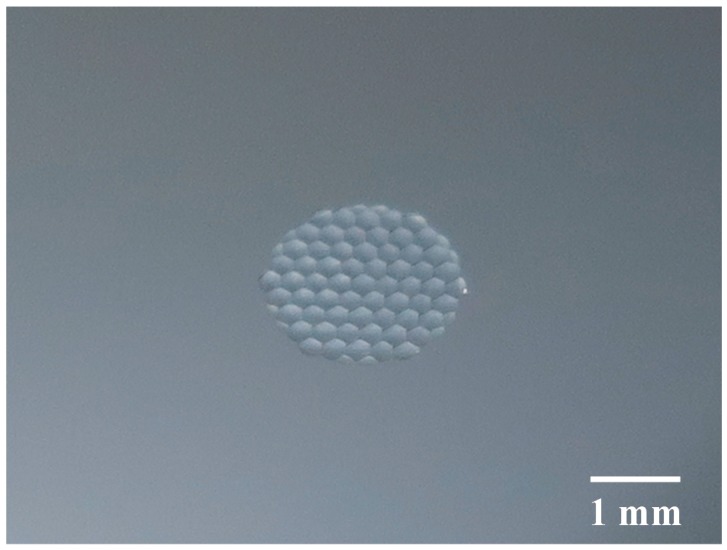
Photograph of a hexagonal microlens array machined on a single-crystal silicon wafer.

**Figure 17:**
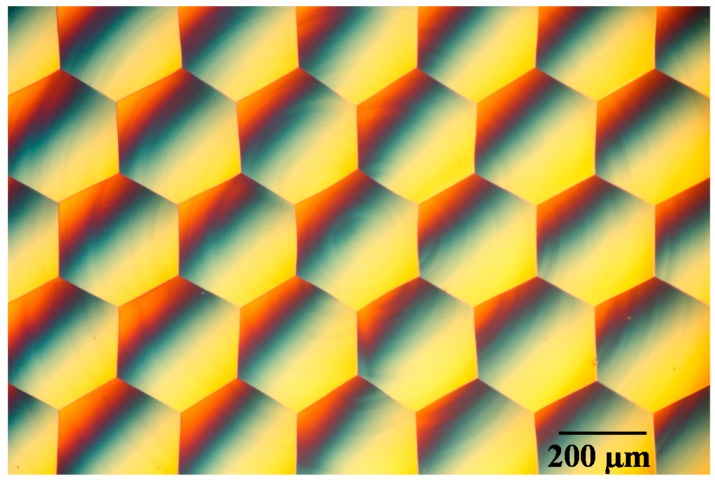
Differential interference contrast microscope image of the hexagonal microlens array.

Figure 16 shows a photograph of a hexagonal microlens array having 58 lenslets machined on a single-crystal silicon wafer (001) plane. It took about 2 h for machining. Figure 17 shows a differential interference contrast microscope image of the hexagonal microlens array. No cracks were found on the machined lenslet surfaces, which means the microlens arrays were successfully machined in a ductile mode. In addition, sharp edges among the lenslets were precisely generated by the segment turning method.

**Figure 18:**
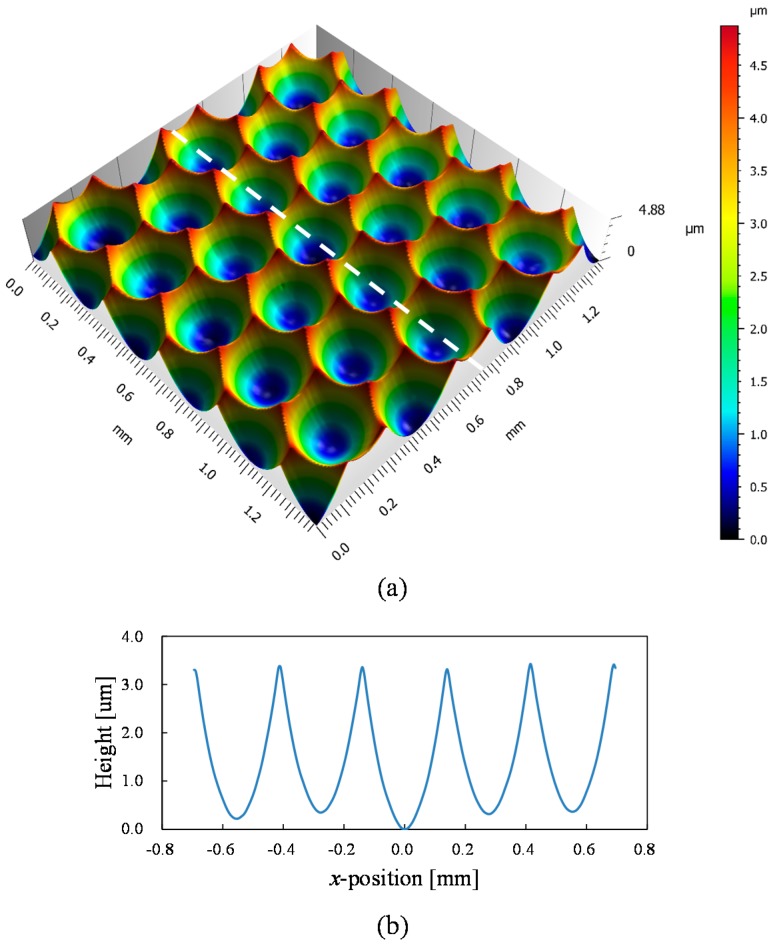
Lens shape measurement results: (**a**) three-dimensional topography of a hexagonal microlens array; and (**b**) cross-sectional profile measured along the white dashed line shown in (**a**).

The surface of the machined microlens array was then measured using a laser-probe profilometer NH-3SP (Mitaka Kouki Co., Ltd., Tokyo, Japan). Figure 18 shows a three-dimensional topography and cross-sectional profile along the *x*-axis shown in Figure 15 (white dashed line shown in Figure 18a). It was confirmed that the lenslets have almost the same depth in the whole lens area.

**Figure 19:**
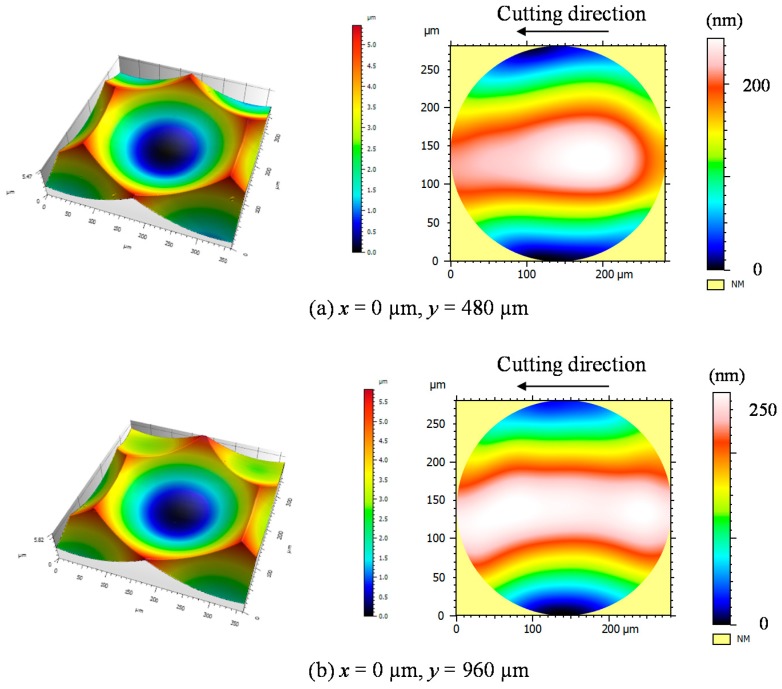
Three-dimensional topographies of lenslets at different locations and their form error distributions.

The lenslet surface was further measured using a white light interferometer Talysurf CCI1000 (AMETEK Taylor Hobson Ltd., Leicester, UK) to evaluate its form error and surface roughness. Figure 19 shows three-dimensional topographies and form error distributions of lenslets the center of which is located at (*x*, *y*) = (0 μm, 480 μm) and (0 μm, 960 μm) as shown in Figure 15. The P-V value of the form error measured was 270 nm (Figure 19a) and 249 nm (Figure 19b). The lenslet surface roughness was 4.8 nmSa (Figure 19a) and 4.9 nmSa (Figure 19b). The surface roughness was measured in a round area (diameter 300 μm) in lenslet centers and calculated by removing the lens curvature and tilt. These results meet the requirement of infrared optical systems, as discussed in our previous paper [16].

### 4.3. Evaluation of Subsurface Damage

**Figure 20:**
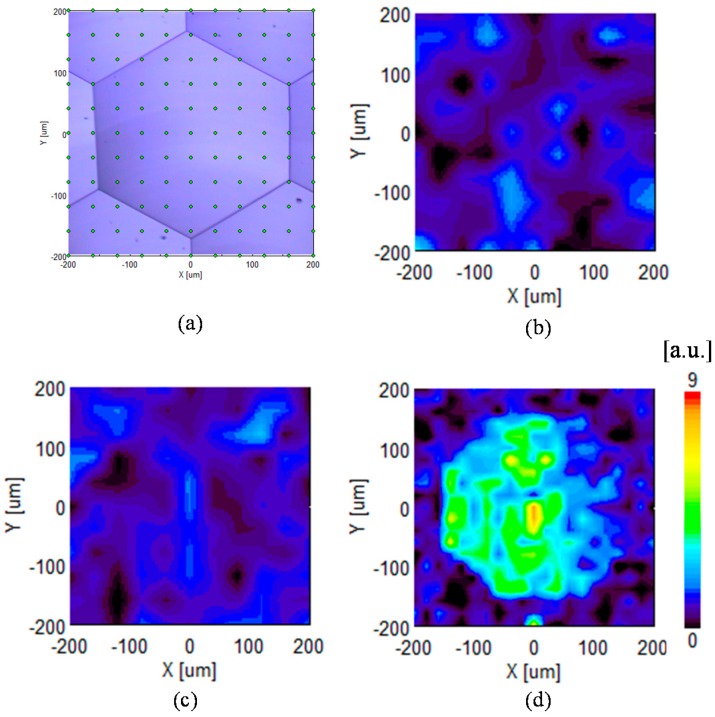
Mapping measurement of peak intensity of amorphous silicon using a laser micro-Raman spectrometer: (**a**) distribution of measurement points; (**b**) mapping result for the lenslet located at *x* = 0 μm, *y* = 480 μm and (**c**) *y* = 960 μm; (**d**) is mapping result for a circular dimple at a higher feed rate (*f* = 6 μm/rev), showing higher intensity.

In general, a subsurface damage layer containing amorphous silicon is generated when cutting single-crystal silicon due to the high pressure from the diamond tool [26]. This subsurface damage layer may affect the optical performance of the resulting lens array, because its optical properties such as transmission rate and refractive index are different from those of single-crystal silicon. To confirm this, laser micro-Raman spectroscopy [27] was performed to evaluate the degree of silicon amorphization on the machined lenslet surface. The excitation wavelength of the laser in Raman spectroscopy was 532 nm. Raman mapping measurements were performed as shown by the dots in Figure 20a. Figure 20b,c show mapping results of the peak intensity for amorphous silicon (470 cm^−1^) for a lenslet located at *y* = 480 μm and 960 μm. Compared with the mapping result for a circular dimple machined at a higher feed rate (*f* = 6 μm/rev) shown in Figure 20d, less amorphous silicon was detected. It is thought that the cutting force induced at a small feed rate was so small that it did not cause significant phase transformation of silicon. Thus, the machined microlens arrays in this study have negligible subsurface damage and maybe used in infrared optical systems without any subsequent processing like polishing.

### 4.4. Tool Observation

**Figure 21:**
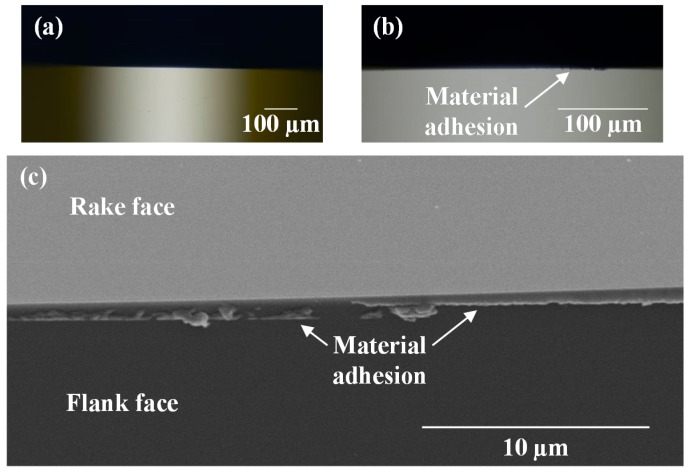
Tool observation results: (**a**) microscope image of tool edge before cutting; (**b**) microscope image and (**c**) Scanning electron microscope (SEM) image of the tool edge after cutting hexagonal microlens arrays on silicon.

The diamond tool was observed using a scanning electron microscope (SEM) after cutting hexagonal microlens arrays for a total cutting distance of 7.88 m. Figure 21 shows microscopic images and an SEM image of the tool edge. Material adhesion was found on the flank face. This phenomenon maybe caused by the decrease of effective relief angle when the tool cuts into a lenslet. An extremely small relief angle leads to squeeze and adhesion of workpiece material onto the tool flank face. It is noteworthy that in Figure 21, no obvious tool wear was observed. This demonstrated that tool wear was insignificant for such a short cutting distance. Especially, in the segment turning method, the tool/workpiece contact is intermittent, which enables the tool to be lubricated and cooled effectively. As a result, tool wear is significantly reduced compared to that in continuous turning [28].

## 5. Conclusions

Segment turning using an STS system was proposed for machining microlens arrays with sharp edges and its effectiveness was experimentally and analytically demonstrated. Hexagonal microlens arrays were successfully fabricated on single-crystal silicon. The following conclusions were obtained.

(1) Compared to continuous turning, the form error of the microlens array fabricated by segment turning was reduced to 24% at the same spindle rotation rate and 76% for the same machining time, respectively. The segment turning method reduced significantly the *Z*-axis acceleration at lenslet boundaries and in turn, eliminated the dynamic errors of the machine tool.
(2) A simplified STS control model was proposed for predicting tool paths and position errors due to machine table acceleration and was experimentally verified.
(3) Hexagonal silicon microlens arrays with a form error of ~300 nm P-V and surface roughness of ~5 nmSa were successfully fabricated.
(4) Hexagonal silicon microlens arrays were machined in a completely ductile mode with sharp edges at the boundaries of lenslets.
(5) Raman spectroscopy of the lenslet surfaces showed that machining-induced amorphization of silicon was reduced, indicating high surface integrity of the fabricated lenses.

## Figures and Tables

**Table 1:** Cutting conditions for a hexagonal microlens array on aluminum alloy.

Cutting Parameters	Values
Depth of cut *Ap* (μm)	10 (roughing cut)
	2 (finishing cut)
Spindle rotation rate *N* (rpm)	15, 45 (continuous STS turning)
	45 (segment STS turning)
Feed per revolution *f* (μm/rev)	1
Cutting speed *Vc* (mm/s)	0~7.33 (*N* = 15)
	0~23.5 (*N* = 45)
Cutting tool	
Tool material	Single-crystal diamond
Nose radius (mm)	0.1
Rake angle (°)	0
Relief angle (°)	6
Coolant	Oil mist

**Table 2:** Identified parameters of the transfer function.

*n* (*d* = 4)	*a_n_*	*b_n_*
1	−1.4590	0.0332
2	−0.1072	−0.0424
3	0.5700	0.0160
4	0.3923	0.0360
5	−0.4839	−0.0609
6	−0.4312	0.0475
7	0.6291	0.0138
8	0.1703	−0.0914
9	−0.3175	0.0993
10	0.0401	−0.0477

**Table 3:** Cutting conditions for a hexagonal microlens array of single-crystal silicon.

Cutting Parameters	Values
Depth of cut *Ap* (μm)	0~6
Spindle rotation rate *N* (rpm)	40
Feed per revolution *f* (μm/rev)	1
Cutting speed *Vc* (mm/s)	0~4.70
Cutting tool	
Tool material	Single-crystal diamond
Nose radius (mm)	1
Rake angle (°)	−30
Relief angle (°)	36
Coolant	Oil mist

## References

[1] Morishita K., Nakajima K., Fujii T., Shiinoki M. (2011). Near-net shaping of single-crystal silicon for optical lens by one-shot pressing at temperature just below silicon melting point and its demonstration of optical properties. Appl. Phys. Express.

[2] Kim W., Matsuhara H., Onaka T., Kataza H., Wada T., Uemizu K., Ueno M., Murakami H., Fujishiro N., Ishihara D. (2005). Optical performance evaluation of Near InfraRed camera (NIR) on board ASTRO-F. Proc. SPIE.

[3] Fletcher D.A., Crozier K.B., Quate C.F., Kino G.S., Goodson K.E. (2000). Near-field infrared imaging with a microfabricated solid immersion lens. Appl. Phys. Lett..

[4] Deng Z., Yang Q., Chen F., Meng X., Bian H., Yong J., Shan C., Hou X. (2015). Fabrication of large-area concave microlens array on silicon by femtosecond laser micromachining. Opt. Lett..

[5] Pan A., Gao B., Chen T., Si J., Li C., Chen F., Hou X. (2014). Fabrication of concave spherical microlenses on silicon by femtosecond laser irradiation and mixed acid etching. Opt. Express.

[6] Nitta T., Naruse M., Sekimoto Y., Mitsui K., Okada N., Karatsu K., Sekine M., Matsuo H., Noguchi T., Uzawa Y. (2013). Beam pattern measurement of millimeter-wave kinetic inductance detector camera with direct machined silicon lens array. IEEE Trans. THz Sci. Technol..

[7] He P., Li L., Li H., Yu J., James Lee L., Yi A.Y. (2014). Compression molding of glass freeform optics using diamond machined silicon mold. Manuf. Lett..

[8] Albero J., Nieradko L., Gorecki C., Ottevaere H., Gomez V., Thienpont H., Pietarinen J., Päivänranta B., Passilly N. (2009). Fabrication of spherical microlenses by a combination of isotropic wet etching of silicon and molding techniques. Opt. Express.

[9] Oliveira O.G., Lima Monteiro D.W., Costa R.F.O. (2014). Optimized microlens-array geometry for Hartmann-Shack wavefront sensor. Opt. Laser Eng..

[10] Ow Y.S., Breese M.B.H., Azimi S. (2010). Fabrication of concave silicon micro-mirrors. Opt. Express.

[11] Weck M., Hennig J., Hilbing R. (2001). Precision Cutting Processes for Manufacturing of Optical Components. Proc. SPIE.

[12] Chen C.C., Huang C.Y., Peng W.J., Cheng Y.C., Yu Z.R., Hsu W.Y. (2013). Freeform surface machining error compensation method for ultra-precision slow tool servo diamond turning. Proc. SPIE.

[13] Fang F.Z., Zhang X.D., Weckenmann A., Zhang G.X., Evans C. (2013). Manufacturing and measurement of freeform optics. CIRP Ann. Manuf. Technol..

[14] Davis G.E., Roblee J.W., Hedegs A.R. (2009). Comparison of freeform manufacturing techniques in the production of monolithic lens arrays. Proc. SPIE.

[15] Scheiding S., Yi A.Y., Gebhardt A., Loose R., Li L., Risse S., Eberhardt R., Tünnermann A. (2011). Diamond milling or turning for the fabrication of micro lens arrays: Comparing different diamond machining technologies. Proc. SPIE.

[16] Mukaida M., Yan J. (2017). Ductile machining of single-crystal silicon for microlens arrays by ultraprecision diamond turning using a slow tool servo. Int. J. Mach. Tools Manuf..

[17] Chou M.C., Pan C.T., Shen S.C., Chen M.F., Lin K.L., Wu S.T. (2005). A novel method to fabricate gapless hexagonal micro-lens array. Sens. Actuators A.

[18] Yu D.P., Hong G.S., Wong Y.S. (2012). Profile error compensation in fast tool servo diamond turning of micro-structured surfaces. Int. J. Mach. Tools Manuf..

[19] Fang F.Z., Zhang X.D., Hu X.T. (2008). Cylindrical coordinate machining of optical freeform surfaces. Opt. Express.

[20] Yin Z.Q., Dai Y.F., Li S.Y., Guan C.L., Tie G.P. (2011). Fabrication of off-axis aspheric surfaces using a slow tool servo. Int. J. Mach. Tools Manuf..

[21] Chen C.C., Cheng Y.C., Hsu W.Y., Chou H.Y., Wang P.J., Tsai D.P. (2011). Slow tool servo diamond turning of optical freeform surface for astigmatic contact lens. Proc. SPIE.

[22] Yi A.Y., Li L. (2005). Design and fabrication of a microlens array by use of a slow tool servo. Opt. Lett..

[23] Zhang X., Fang F., Yu L.H., Jiang L., Guo Y. (2013). Slow slide servo turning of compound eye lens. Opt. Eng..

[24] Yu D.P., Hong G.S., Wong Y.S. (2011). Integral sliding mode control for fast tool servo diamond turning of micro-structured surfaces. Int. J. Autom. Technol..

[25] Hjalmarsson H. (2005). From experiment design to closed-loop control. Automatica.

[26] Yan J., Asami T., Harada H., Kuriyagawa T. (2009). Fundamental investigation of subsurface damage in single crystalline silicon caused by diamond machining. Precis. Eng..

[27] Yan J. (2004). Laser micro-Raman spectroscopy of single-point diamond machined silicon substrates. Appl. Phys..

[28] Yan J., Syoji K., Tamaki J. (2003). Some observations on the wear of diamond tools in ultra-precision cutting of single-crystal silicon. Wear.

